# Tat-antioxidant 1 protects against stress-induced hippocampal HT-22 cells death and attenuate ischaemic insult in animal model

**DOI:** 10.1111/jcmm.12513

**Published:** 2015-03-17

**Authors:** So Mi Kim, In Koo Hwang, Dae Young Yoo, Won Sik Eum, Dae Won Kim, Min Jea Shin, Eun Hee Ahn, Hyo Sang Jo, Eun Ji Ryu, Ji In Yong, Sung-Woo Cho, Oh-Shin Kwon, Keun Wook Lee, Yoon Shin Cho, Kyu Hyung Han, Jinseu Park, Soo Young Choi

**Affiliations:** a Department of Biomedical Science and Research Institute of Bioscience and Biotechnology, Hallym UniversityChunchon, Korea; b Department of Anatomy and Cell Biology, College of Veterinary Medicine, and Research Institute for Veterinary Science, Seoul National UniversitySeoul, Korea; c Department of Biochemistry and Molecular Biology, Research Institute of Oral Sciences, College of Dentistry, Kangnung-Wonju National UniversityKangneung, Korea; d Department of Biochemistry and Molecular Biology, University of Ulsan College of MedicineSeoul, Korea; e Department of Biochemistry, School of Life Sciences & Biotechnology, Kyungpook National UniversityTaegu, Korea

**Keywords:** Tat-Atox1, ischaemic injury, oxidative stress, protein transduction domain, protein therapy

## Abstract

Oxidative stress-induced reactive oxygen species (ROS) are responsible for various neuronal diseases. Antioxidant 1 (Atox1) regulates copper homoeostasis and promotes cellular antioxidant defence against toxins generated by ROS. The roles of Atox1 protein in ischaemia, however, remain unclear. In this study, we generated a protein transduction domain fused Tat-Atox1 and examined the roles of Tat-Atox1 in oxidative stress-induced hippocampal HT-22 cell death and an ischaemic injury animal model. Tat-Atox1 effectively transduced into HT-22 cells and it protected cells against the effects of hydrogen peroxide (H_2_O_2_)-induced toxicity including increasing of ROS levels and DNA fragmentation. At the same time, Tat-Atox1 regulated cellular survival signalling such as p53, Bad/Bcl-2, Akt and mitogen-activate protein kinases (MAPKs). In the animal ischaemia model, transduced Tat-Atox1 protected against neuronal cell death in the hippocampal CA1 region. In addition, Tat-Atox1 significantly decreased the activation of astrocytes and microglia as well as lipid peroxidation in the CA1 region after ischaemic insult. Taken together, these results indicate that transduced Tat-Atox1 protects against oxidative stress-induced HT-22 cell death and against neuronal damage in animal ischaemia model. Therefore, we suggest that Tat-Atox1 has potential as a therapeutic agent for the treatment of oxidative stress-induced ischaemic damage.

## Introduction

Antioxidant 1 (Atox1) protein, known as ATX1, consists of 68 amino acids and is a copper chaperone which plays a crucial role in copper homoeostasis. Copper, essential for all living organisms, is known to be an important catalytic co-factor involved in many biochemical processes including respiration, antioxidant defence and iron metabolism in eukaryotic cells 1–4. Atox1 protein activates ceruloplasmin by delivering cytosolic copper to the ATPase protein in the trans-Golgi network 5. Some studies have demonstrated that Atox1 protein protects cells against superoxide and hydrogen peroxide (H_2_O_2_)-induced toxicity 6–8. In addition, others studies have demonstrated that the oxidative protection of Atox1 is dependent on both copper-binding residue in the N-terminal domain and lysine residue in the C-terminal domain. However, the copper chaperone function of Atox1 is dependent on copper-binding residue in the N-terminal domain. In addition, Atox1 utilizes a copper-dependent transcription factor to increase promoter activity of cyclin D1 and SOD3, important regulators of cell cycle G_1_-S phase progression, which also promote cell proliferation. Thus, the results of this study suggest that Atox1 is a therapeutic target for various diseases such as cardiovascular disease, cancer, and Wilson's disease 1,3,9,10.

Reactive oxygen species (ROS) are a natural by-product of the normal cellular metabolism of oxygen. An imbalance in ROS can result in abnormal structural and functional properties resulting in damage to cellular components including DNA and proteins 11. Elevated ROS production during cellular metabolism is associated with different pathological processes and leads to various diseases as well as to cell death 12. Several studies have focused on ROS induced by the interruption and reperfusion of blood flow to the brain 13,14. A steep increase in ROS production in the hippocampal CA1 region leads to neuronal apoptosis. It is therefore assumed that ROS management has protective effects against ischaemic injury to the brain 15.

Protein transduction technology is an effective method to deliver proteins into mammalian cells. Protein transduction domains (PTDs), also called cell-penetrating peptides, are commonly used to transport exogenous proteins into cells. Although the precise mechanism of this delivery is unclear, PTDs are commonly used to deliver therapeutic proteins *in vitro* and *in vivo*. Tat peptide, HIV-1 Tat transduction domain, is the most common PTD 16–19. In our previous studies, we have reported on the protective effects of various transduced PTDs fusion protein against cell death *in vitro* and *in vivo* 20–24.

This study was aimed to investigate the functions of transduced Tat-Atox1 protein on oxidative stress-induced hippocampal HT-22 cell death and transient forebrain ischaemia. The findings led to the conclusion that transduced Tat-Atox1 protein demonstrated protective effects against oxidative stress in *in vitro* and *in vivo* suggesting potential therapeutic efficacy of Tat-Atox1 protein for the treatment of not only transient forebrain ischaemia but also other oxidative stress-associated neuronal disorders.

## Materials and methods

### Cell culture and materials

HT-22, mouse hippocampal cells were grown in DMEM containing 10% foetal bovine serum and antibiotics (100 μg/ml streptomycin, 100 U/ml penicillin) at 37°C in a humidity chamber with 5% CO_2_ and 95% air. Ni^b+^- → Ni^2+^- nitrilotriacetic acid Sepharose superflow was purchased from Qiagen (Valencia, CA, USA). PD-10 columns were purchased from Amersham (Brauncschweig, Germany). The indicated primary and β-actin antibodies were obtained from Cell Signaling Technology (Beverly, MA, USA) and Santa Cruz Biotechnology (Santa Cruz, CA, USA). Tat peptides were purchased from PEPTRON (Daejeon, Korea). Unless otherwise stated, all other agents were of the highest grade available.

### Purification and transduction of Tat-Atox1 proteins into HT-22 cells

Preparation of the Tat expression vector has been described in a previous study 25. Human Atox1 was amplified by PCR with two primers. The sense primer 5′-CTCGAGATGCCGAAGCACG-3′ contained an *Xho*I restriction site. The antisense primer 5′-GGATCCCTACTCAAGGCCAAGG-3′ contained a *Bam*HI restriction site. The resulting PCR products were ligated into the TA vector and cut with *Xho*I and *Bam*HI. Fragments were then ligated into the Tat expression vector to generate Tat-Atox1. Control Atox1 was manufactured without the Tat peptide. Recombinant Tat-Atox1 plasmid was transformed into *Escherichia coli* BL21 (DE3) and cultured in 0.5 mM isopropyl-β-d-thio-galactoside (Duchefa, Haarlem, the Netherlands) at 18°C for over 24 hrs. Harvested cells were lysed by sonication and Tat-Atox1 protein was purified using a Ni^b+^- → Ni^2+^- nitrilotriacetic acid Sepharose affinity column and PD-10 column chromatography to generate Tat-Atox1 protein. Bovine serum albumin was used as a standard and protein concentration was measured by Bradford assay 26.

To examine time and concentration dependent transduction ability of Tat-Atox1 protein, HT-22 cells were exposed to different concentration (0.5–3 μM) of Tat-Atox1 protein and Atox1 protein for 1 hr and to 3 μM for various time periods (10–60 min.). Cells were then washed with PBS and treated with trypsin-EDTA. The amounts of transduced proteins were measured by Western blotting. Also, the intracellular stability of Tat-Atox1 protein was examined after being harvested at various times (1–36 hrs) using a rabbit anti-polyhistidine antibody (Santa Cruz Biotechnology).

### Western blot analysis

Equal amounts of proteins were analysed using 15% SDS-PAGE. Analysed proteins were electrotransferred to a nitrocellulose membrane, and the membrane was blocked with TBS-T (25 mM Tris-HCl, 140 mM NaCl, 0.1% Tween 20, pH 7.5) buffer containing 5% non-fat dry milk. The membrane was analysed by Western blot using primary antibodies recommended by the manufacturer. Proteins were identified using chemiluminescent reagents as recommended by the manufacturer (Amersham, Franklin Lakes, NJ, USA) 27.

### Confocal fluorescence microscopy

To determine the intracellular distribution of transduced Tat-Atox1 protein in HT-22 cells, we performed confocal fluorescence microscopy as described previously 27. Culture media were placed on coverslips and treated with 3 μM Tat-Atox1 protein. After 1 hr of incubation at 37°C, the cells were washed with PBS twice and fixed with 4% paraformaldehyde for 5 min. The cells were treated in PBS containing 3% bovine serum albumin, 0.1% Triton X-100 (PBS-BT) at room temperature for 30 min. and washed with PBS-BT. The primary antibody (His-probe, Santa Cruz Biotechnology) was diluted 1:2000 and incubated at room temperature for 4 hrs. The secondary antibody (Alexa fluor 488; Invitrogen, Carlsbad, CA, USA) was diluted 1:15,000 and incubated in the dark for 1 hr. Nuclei were stained with 1 μg/ml DAPI (Roche Applied Science, Mannheim, Germany) for 2 min. Stained cells were analysed using a confocal fluorescence microscope confocal laser-scanning system (Bio-Rad MRC-1024ES, 4BIOROD, CA, USA).

### 3-(4,5-dimethylthiazol-2-yl)-2,5-dipheyltetrazolium bromide (MTT) assay

The biological activity of Tat-Atox1 protein was measured by assessing cell viability after exposure to H_2_O_2_ as described previously 21,27. HT-22 cells were plated at a confluence of 70% in a 96 well plate and exposed to Tat-Atox1 proteins and Atox1 proteins (0.5–3 μM). After 1 hr, cells were treated with 1 mM H_2_O_2_ for 2 hrs. Cell viability was measured at 540 nm using an ELISA microplate reader (Labsystems Multiskan MCC/340, Helsinki, Finland) and cell viability was expressed as a percentage of untreated control cells.

### Measurement of intracellular ROS levels

Intracellular ROS levels were measured using 2′,7′-dichlorofluorescein diacetate (DCF-DA), which converts to fluorescent DCF in cells when exposed to ROS as described previously 21,27. ROS levels were measured in HT-22 cells in the presence or absence of Tat-Atox1 protein (0.5–3 μM). After 1 hr of pre-treatment with Tat-Atox1 protein, the cells were treated with H_2_O_2_ (1 mM) for 10 min. After being washed with PBS, the cells were treated with DCF-DA at a dose of 20 μM for 30 min. Fluorescence levels were measured using a Fluoroskan ELISA plate reader (Labsystems Oy, Helsinki, Finland) at 485 nm excitation and 538 nm emission.

### TUNEL assay

To examine whether transduced Tat-Atox1 proteins protect against DNA damage in H_2_O_2_-treated cells, HT-22 cells were pre-treated with 3 μM Tat-Atox1 protein for 1 hr and exposed to 1 mM H_2_O_2_ for 4 hrs. Terminal deoxynucleotidyl transferase-mediated biotinylated dUTP nick end labelling (TUNEL) and a Cell Death Detection kit (Roche Applied Science) were used to assess cellular damage. Images were analysed using a fluorescence microscope (Nikon eclipse 80i, Tokyo, Japan) 21,27. Fluorescence levels were measured using a Fluoroskan ELISA plate reader (Labsystems Oy) at 485 nm excitation and 538 nm emission.

### Experimental animals and treatment

Gerbils were housed at an adequate temperature (23°C) and humidity (60%) with a 12 hrs light/12 hrs dark cycle, and free access to food and water. All experimental procedures involving animals and their care conformed to the Guide for the Care and Use of Laboratory Animals of the National Veterinary Research and Quarantine Service of Korea and were approved by the Hallym Medical Center Institutional Animal Care and Use Committee.

The ischaemia animal model was carried out as previously described 21,27. To explore the protective effects of Tat-Atox1 protein against ischaemic damage, the animals were divided into five groups (each *n* = 10); control sham group, vehicle group, Tat peptide-, control Atox1 protein and Tat-Atox1 protein-treated groups (3 mg/kg). Tat peptide, control Atox1 protein and Tat-Atox1 protein were intraperitoneally injected 30 min. after reperfusion.

### Immunohistochemistry and protective effects of Tat-Atox1

Immunohistochemistry was performed as previously described 27–29. Briefly, the sections were incubated with diluted mouse anti-neuronal nuclei (NeuN, 1:1000; Chemicon International, Temecula, CA, USA), rabbit anti-glial fibrillary acidic protein (GFAP, 1:1000; Chemicon International) and rabbit anti-ionized calcium-binding adapter molecule 1 (Iba-1, 1:500; Wako, Osaka, Japan) for 48 hrs at 4°C. Thereafter, they were exposed to biotinylated goat anti-rabbit IgG or goat antimouse IgG, streptavidin peroxidase complex (diluted 1:200; Vector Laboratories, Burlingame, CA, USA), and then visualized with 3,3′-diaminobenzidine tetrahydrochloride (Sigma-Aldrich Co., St. Louis, MO, USA) in 0.1 M Tris-HCl buffer (pH 7.4).

To examine the neuroprotective effect of Tat-Atox1 protein in an animal ischaemia model, we measured spontaneous motor activity and lipid peroxidation as described previously 30,31. Spontaneous motor activity was monitored over 24 hrs while simultaneously the number of times each animal reared and the time (in seconds) spent in grooming behaviour were recorded. Each animal was observed continuously *via* a 4 × 8 photobeam. Scores were generated from live observations, and video sequences were used for subsequent re-analysis.

Also, the 4-hydroxy-2-nonenal (HNE) level, which has been used as an indicator of lipid peroxidation in hippocampal tissues, was measured using an HNE assay kit. Control and ischaemia-operated animals (*n* = 5 in each group) were anaesthetized with 30 mg/kg Zoletil 50 at 3 hrs after reperfusion. Then the hippocampi were removed for measurement of HNE using a Bioxytech HAE-586 spectrophotometric assay kit (OxisResearch, Portland, OR, USA). This was carried out 3 hrs after reperfusion because lipid peroxidation peaked at this time-point. Hippocampi were homogenized in a buffer containing 10 mM HEPES (pH 7.5) containing 200 mM mannitol, 70 mM sucrose, 1 mM EGTA and 5 mM butylated hydroxytoluene. One millilitre of homogenate was extracted with dichloromethane, and an aliquot of the lower organic phase was dried and reconstituted with water. The sample was mixed with *N*-methyl-2-phenylindole in acetonitrile and methanesulfonic acid, after which the mixture was incubated and centrifuged. The absorbance of the clear supernatant was determined at 586 nm using a Beckman DU-64 spectrophotometer (Beckman, Fullerton, CA, USA). The HNE concentrations in experimental samples were determined against HNE standards provided in the assay kit.

### Quantitative and statistical analysis

The measurement of NeuN-, GFAP- and Iba-1-immunoreactive cells in all groups was performed with an image analysis system equipped with a computer-based CCD camera (software: Optimas 6.5; CyberMetrics, Scottsdale, AZ, USA). In addition, images of all NeuN-immunoreactive structures were taken from the hippocampal CA1 region through a BX51 light microscope (Olympus, Tokyo, Japan) equipped with a digital camera (DP71; Olympus) connected to a computer monitor. NeuN-immunoreactive cells in each section of the hippocampal CA1 regions were then counted using Optimas 6.5 software (CyberMetrics). The cell counts from all sections of all gerbils were averaged.

In addition, analysis of a region of interest in the hippocampal CA1 region was performed with an image analysis system. Images were calibrated into an array of 512 × 512 pixels corresponding to a tissue area of 140 × 140 μm (40× primary magnification). Each pixel resolution was set to 256 grey levels. The intensity of GFAP and Iba-1 immunoreactivity was evaluated by means of relative optical density (ROD), which was obtained after the transformation of the mean grey level using the formula: ROD = log (256/mean grey level). ROD of background was determined in unlabelled portions and the value subtracted for correction, yielding high ROD values in the presence of preserved structures and low values after structural loss using NIH Image 1.59 software, National Institute of Mental Health, Bethesda, Maryland, USA. A ratio of the ROD was calibrated as %.

Data are expressed as the mean ± SEM of three experiments. Differences between groups were analysed by anova followed by a Bonferroni's *post hoc* test. Statistical significance was considered at *P *<* *0.05.

## Results

### Expression and purification of Tat-Atox1 protein

**Figure 1:**
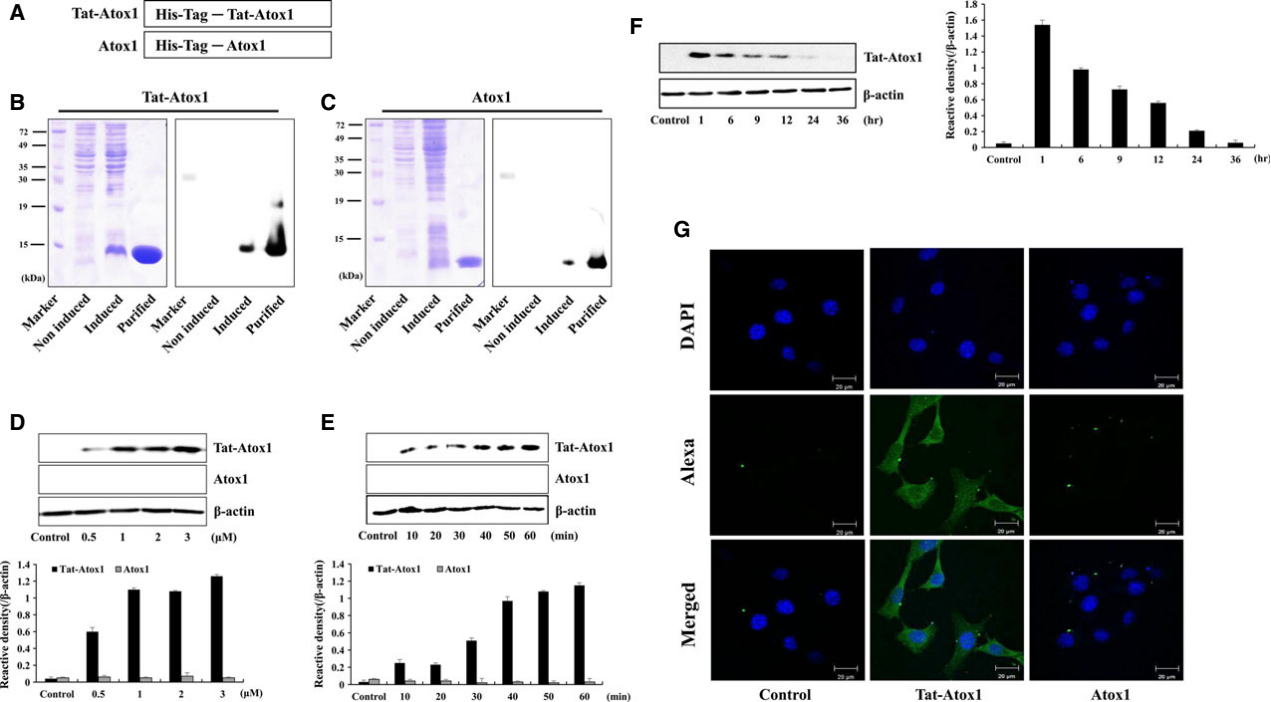
Purification and transduction of Tat-Atox1 protein into HT-22 cells. Overview of Tat-Atox1 protein (A). Expression and purification of Tat Atox1 protein (B) and control Atox1 protein (C) were detected by Western blot analysis using 15% SDS-PAGE and rabbit anti-polyhistidine antibody. Tat-Atox1 and control Atox1 proteins (0.5–3 μM) were added to the culture media for 1 hr (D), Tat-Atox1 and control Atox1 proteins (3 μM) were added to the culture media for 10–60 min. (E), Tat-Atox1 protein (3 μM) was transduced into cells for 1 hr and the cells were incubated for 36 hrs (F). Then the cells were treated with trypsin-EDTA, washed with PBS three times. Transduction of Tat-Atox1 protein was measured by Western blotting, and the intensity of the bands was measured by a densitometer. The localization of transduced Tat-Atox1 protein (G). After transduction of Tat-Atox1 protein (3 μM) for 1 hr, the localization of transduced Tat-Atox1 protein was examined by confocal fluorescence microscopy; scale bar = 20 μm.

Human Atox1 genes were fused to a Tat peptide expression vector to produce cell-penetrating Tat-Atox1 proteins. Control Atox1 protein was also manufactured in the absence of Tat PTD (Fig.1A). Tat-Atox1 proteins were purified following overexpression using a Ni^b+^- → Ni^2+^- nitrilotriacetic acid Sepharose affinity column and PD-10 column chromatography. Purified Tat-Atox1 proteins were identified by SDS-PAGE and Western blotting. Figure1B and C show the results of Western blotting performed with a rabbit anti-polyhistidine antibody following the expression and purification of Tat-Atox1 and control Atox1 proteins on a 15% SDS-PAGE.

### Transduction of Tat-Atox1 protein into HT-22 cell

To examine Tat-Atox1 protein transduction ability into HT-22 cells, HT-22 cells were treated with various concentrations of Tat-Atox1 proteins over various time periods. Transduction of Tat-Atox1 protein into HT-22 cells was assessed at different concentrations (0.5–3 μM) for 60 min. using Western blotting. Results indicated that Tat-Atox1 protein transduced into HT-22 cells in a dose-dependent manner (Fig.1D). Also, the cells were treated with 3 μM Tat-Atox1 protein at varying time intervals (10–60 min.) to see whether transduction is time dependent (Fig.1E). Tat-Atox1 protein transduced into the cells gradually increasing for 1 hr. As indicated in Figure1D and E, transduction of Tat-Atox1 protein occurred in a dose- and time-dependent manner whereas control Atox1 protein did not transduce into the cells.

Intracellular stability of transduced Tat-Atox1 proteins in HT-22 cells was also examined. Figure1F shows the stability of transduced Tat-Atox1 protein in HT-22 cells. Tat-Atox1 protein (3 μM) was added to the culture media for different time periods and transduction was measured by Western blotting. Intracellular transduced Tat-Atox1 protein levels were detected after 1 hr. Transduced Tat-Atox1 protein gradually disappeared. However, significant levels of intracellular transduced Tat-Atox1 protein persisted in the cells for 12 hrs.

In addition, we further confirmed the intracellular distribution of the transduced Tat-Atox1 protein by assessing DAPI- and Alexa Fluor 488-stained cells with a confocal fluorescence microscope. As shown in Figure1G, cells treated with transduced Tat-Atox1 protein demonstrated fluorescence in the cytoplasm and nucleus of the cells. However, there was no fluorescence in control Atox1 protein-treated cells. These results indicate that Tat-Atox1 protein efficiently transduced into HT-22 cells and persisted in the cells for 12 hrs after transduction.

### Effects of transduced Tat-Atox1 protein on cell viability in response to oxidative stress

**Figure 2:**
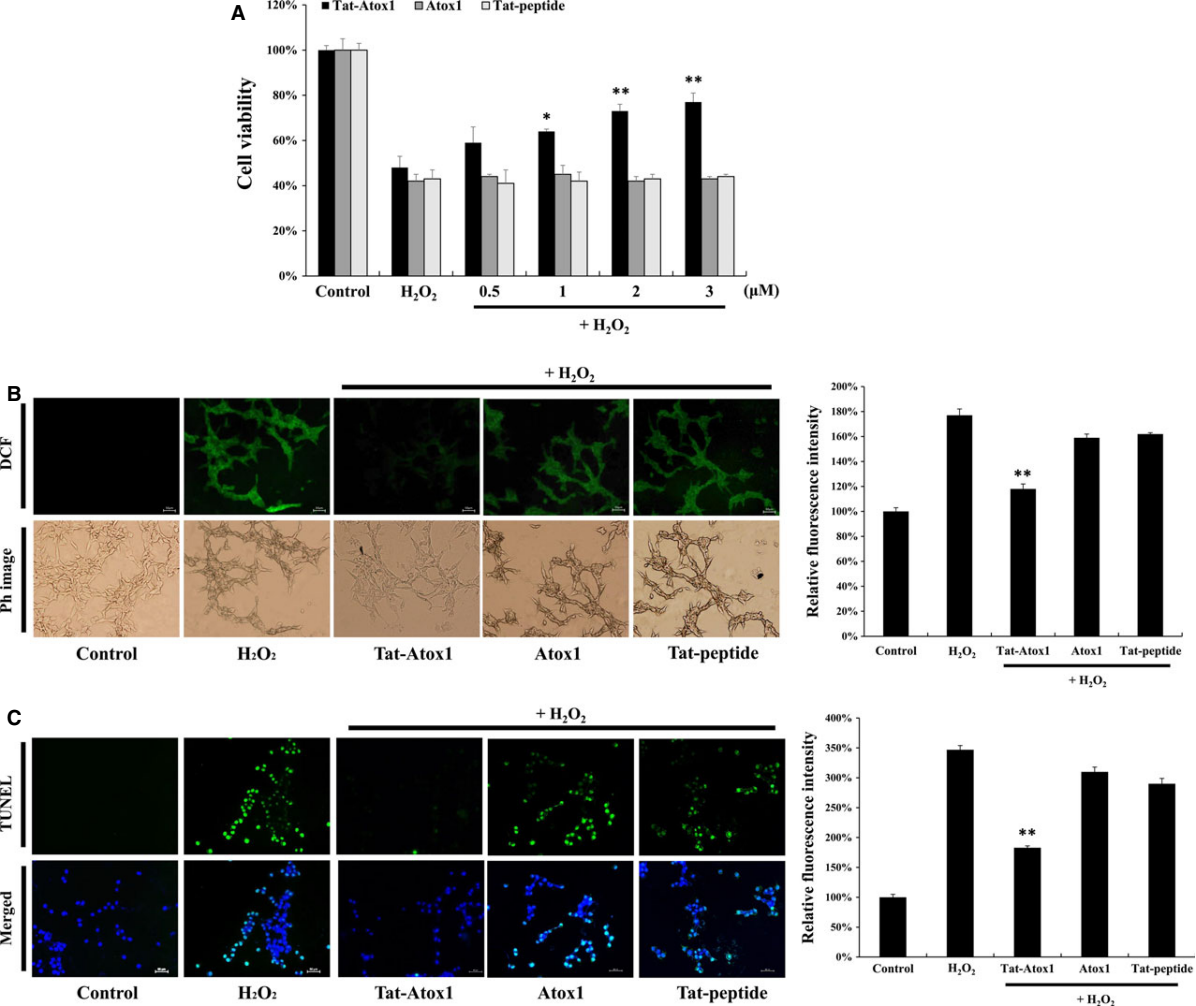
Protective effects of transduced Tat-Atox1 protein against oxidative stress. Pre-treatment of HT-22 cells with Tat-Atox1 protein (0.5–3 μM) and control Atox1 protein for 1 hr and treatment with 1 mM hydrogen peroxide (H_2_O_2_) for 2 hrs. Then, cell viability was assessed by MTT assay (A). *P < 0.05 and **P < 0.01 compared with H_2_O_2_-treated cells. Effects of transduced Tat-Atox1 protein on H_2_O_2_-induced ROS production. Treatment with Tat-Atox1 protein (3 μM) and control Atox1 protein was followed by 10 min. treatment with H_2_O_2_ (1 mM). Intracellular ROS levels were measured by DCF-DA staining and fluorescence intensity was measured by ELISA plate reader (B); scale bar = 50 μm. **P <* *0.01 compared with H_2_O_2_-treated cells. Effects of transduced Tat-Atox1 protein on H_2_O_2_-induced DNA fragmentation. One-hour pre-treatment of HT-22 cells with Tat-Atox1 protein (3 μM) and control Atox1 protein was followed with 4-hr treatment with H_2_O_2_ (1 mM). DNA fragmentation was measured by TUNEL staining and the fluorescent intensity was measured by ELISA plate reader (C); scale bar = 50 μm. **P <* *0.01 compared with H_2_O_2_-treated cells.

To determine whether transduced Tat-Atox1 protein has biological activity under oxidative stress, cells were exposed to H_2_O_2_ and cell viability was examined using a MTT assay. As shown in Figure2A, only 43% of cells exposed to 1 mM of H_2_O_2_ survived. Control Atox1 protein and Tat peptide exhibited no protective effects under the same conditions. However, the cell survival rate of Tat-Atox1 protein-treated cells was markedly increased, up to 78% compared to H_2_O_2_-treated cells and control Atox1 protein-treated cells.

We also determined whether transduced Tat-Atox1 protein inhibited ROS generation, *via* DCF-DA staining. Figure2B represents ROS levels measured in H_2_O_2_-treated cells. After H_2_O_2_ treatment, ROS-stimulated cells were stained with DCF-DA and fluorescence was examined. ROS levels were similar in cells pre-treated with Tat peptide, control Atox1 protein and H_2_O_2_ alone. On the other hand, ROS levels significantly decreased in the Tat-Atox1 protein-treated cells.

The effects of transduced Tat-Atox1 protein on DNA fragmentation were assessed by TUNEL staining. As presented in Figure2C, a greater amount of fluorescence was exhibited in H_2_O_2_-treated cells, compared with control cells. The amount of fluorescence was significantly reduced in the Tat-Atox1 protein-treated cells. However, the same change in fluorescence was not observed in the control Atox1-treated cells. These results indicate that transduced Tat-Atox1 protein has biological activity and protected the cells against oxidative stress *via* its antioxidant role.

### Protective mechanism of Tat-Atox1 on oxidative stress-induced cell death

**Figure 3:**
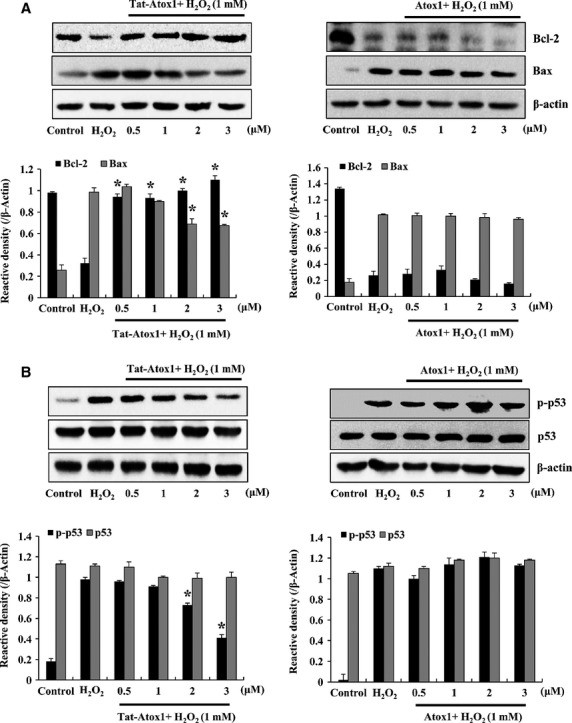
Effects of Tat-Atox1 protein on H_2_O_2_-induced pro- and anti-apoptotic proteins. One-hour pre-treatment of HT-22 cells with Tat-Atox1 protein (0.5–3 μM) and control Atox1 protein was followed with treatment with H_2_O_2_ (1 mM) for 30 min. (Bcl-2/Bax) and 20 min. (p-p53/p53), respectively. The expression levels of Bcl-2/Bax (A) and p-p53/p53 (B) were determined by Western blot analysis and the band intensity was measured by densitometer. (*P < 0.01 compared with H_2_O_2_-treated cells).

Chronic exposure to oxidative stress leads to apoptosis 32,33. To investigate the antioxidant effects of Tat-Atox1 protein on H_2_O_2_-induced apoptotic processes, we tested antioxidant responses of Tat-Atox1 protein on ROS-induced apoptosis using pro- and anti-apoptotic marker proteins. After H_2_O_2_ treatment, the expression of anti-apoptotic protein Bcl-2, and pro-apoptotic protein Bax were measured by Western blotting. The level of Bcl-2 increased in the Tat-Atox1-treated cells whereas Bcl-2 levels exhibited almost no changes in the control Atox1-treated cells. Also, Bax levels were significantly inhibited in the Tat-Atox1-treated cells, however, this protein was not inhibited in the control Atox1-treated groups (Fig.3A).

There are multiple mechanisms by which oxidative stress-derived ROS induces apoptosis and DNA damage. H_2_O_2_-induced overexpression of p53 eventually leads to cell death 34,35. We assessed the anti-apoptotic effects of Tat-Atox1 protein on oxidative stress. The expression of p-p53 was significantly reduced in the Tat-Atox1 protein-treated cells. However, no effect was observed in the control Atox1-treated cells (Fig.3B). These results indicate that Tat-Atox1 protein has anti-apoptotic activity by regulating the Bcl-2, Bax and p-p53 expression levels.

**Figure 4:**
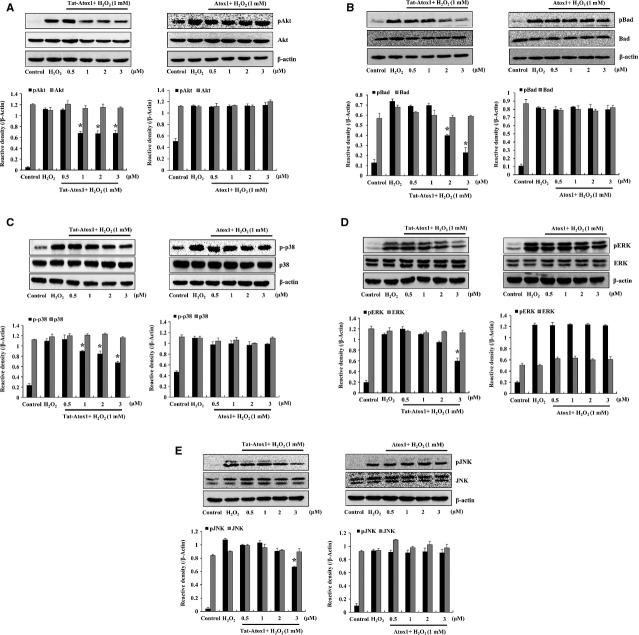
Effects of Tat-Atox1 protein on oxidative stress-induced pAkt/Akt (ser-473), pBad/Bad (s112) and MPPKs. Pre-treatment of HT-22 cells with Tat-Atox1 protein (0.5–3 μM) and control Atox1 protein was followed with treatment with H_2_O_2_ (1 mM) for 10 min. (pAkt/Akt) and 10 min. (pBad/Bad), respectively. H_2_O_2_-induced pAkt/Akt (A) and pBad/Bad (B) expression levels were determined by Western blot analysis and the band intensity was measured by densitometer. The cells were stimulated with H_2_O_2_ (1 mM) for 30 min. with or without pre-treated with Tat-Atox1 protein (0.5–3 μM) for 1 hr. Then, the cells were prepared and analysed for phosphorylation of p38 (C), ERK (D), JNK (E) levels by Western blotting and the band intensities were measured by densitometer. (*P < 0.01 compared with H_2_O_2_-treated cells).

Akt plays a critical role on cell survival and protects against apoptosis. Oxidative stress-induced phosphorylation of Akt (ser-473) activates apoptosis and subsequent neuronal cell death 36. Phosphorylated Akt triggers phosphorylation of the pro-apoptotic protein Bad (s112) 37. Furthermore, several studies have demonstrated that cytoprotection of H_2_O_2_ pre-conditioning against oxidative stress-induced cellular injury is mediated by activation of PI3K/Akt signalling pathway in various cell types 38–40. Thus, we examined the effects of Tat-Atox1 protein on ROS-induced pAkt (s473) and pBad (s112) in H_2_O_2_-treated cells. pAkt and pBad were gradually reduced in the Tat-Atox1 protein-treated cells, while the two proteins remained unchanged in the control Atox1 protein group (Fig.4A and B). These results confirmed the antioxidant defence of Tat-Atox1 protein against oxidative stress-induced apoptosis in H_2_O_2_-stimulated HT-22 cells.

Cell survival signalling is activated by external stress and growth factors such as H_2_O_2_. Among those stimuli, Mitogen-activate protein kinases (MAPKs) play very important roles in ischaemic injuries 41–43. We examined the effect of Tat-Atox1 against the ROS-induced activation of MAPKs. Western blot results demonstrate that the activation of p38 was reduced in a dose-dependent manner in H_2_O_2_-stimulated cells after pre-treatment with Tat-Atox1 protein (Fig.4C). Tat-Atox1 protein also demonstrated inhibitory effects on stress-activated protein kinase/c-Jun N-terminal kinase (SAPK/JNK) and extracellular signal-regulated kinase (ERK) in response to oxidative stress (Fig.4D and E). However, control Atox1 protein did not affect the activation levels of p38, JNK and ERK. Therefore, these results indicate that Tat-Atox1 protein protects against H_2_O_2_-stimulated cell death *via* regulation of MAPKs activation.

### Protective effects of Tat-Atox1 protein against ischaemic damage

**Figure 5:**
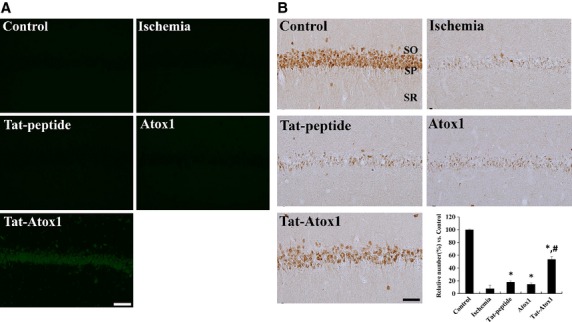
Effect of transduced of Tat-Atox1 protein on animal brain. Transduction of Tat-Atox1 protein into brain (A). Animals were treated with single i.p. injection of Tat-Atox1 protein and killed after 6 hrs. Transduction of Tat-Atox1 protein into the CA1 region was determined by immunohistochemistry with a rabbit anti-polyhistidine antibody and FITC-conjugated anti-rabbit IgG. Immunohistochemistry for NeuN in the hippocampal CA1 region (B). Control, ischaemia, Tat peptide, Atox1 protein and Tat-Atox1 protein-treated groups 4 days after ischaemia-reperfusion. SP, stratum pyramidale; SO, stratum oriens; SR, stratum radiatum; bar = 50 μm. The relative number of NeuN-immunoreactive neurons *versus* control group per section in all the groups (*n* = 5 per group; *P < 0.05, significantly different from the control group, ^*#*^*P* < 0.05, significantly different from the ischaemia group). The bars indicate standard error of mean (SEM).

To assess the protective effects of Tat-Atox1 on ischaemic injury, the transduction of Tat-Atox1 protein into the hippocampal CA1 region of gerbils, crossing the blood-brain barrier (BBB), was measured by immunohistochemial analysis using rabbit anti-polyhistidine antibody and visualized with an FITC-conjugated anti-rabbit IgG (1:200; Jackson ImmunoResearch, West Grove, PA, USA). Figure5A shows the localization of Tat-Atox1 protein in the hippocampal CA1 region.

To determine the neuronal cell viability of transduced Tat-Atox1 proteins in animal ischaemia models, we performed NeuN immunohistochemistry. A large number of NeuN-immunoreactive neurons were observed in the hippocampal CA1 region in the untreated control cells. However, a small number of NeuN-immunoreactive neurons were observed in ischaemia-induced cells, accounting for 7.9% of total numbers observed in the control group (Fig.5B). A small number of NeuN-immunoreactive neurons were found in the cells treated with Tat peptide or Atox1 protein. The number of NeuN-immunoreactive neurons in the Tat peptide- or Atox1 protein-treated groups accounted for 17.9% and 14.2%, respectively, of the total in the control groups. A greater number of NeuN-immunoreactive neurons were observed in the hippocampal CA1 region of the Tat-Atox1 protein-treated ischaemic group, compared with the vehicle-treated ischaemia group. Because, the proportion of NeuN-immunoreactive neurons reached 53.7% of the total number of neurons in the control group, protective action against ischaemic injury was evident.

**Figure 6:**
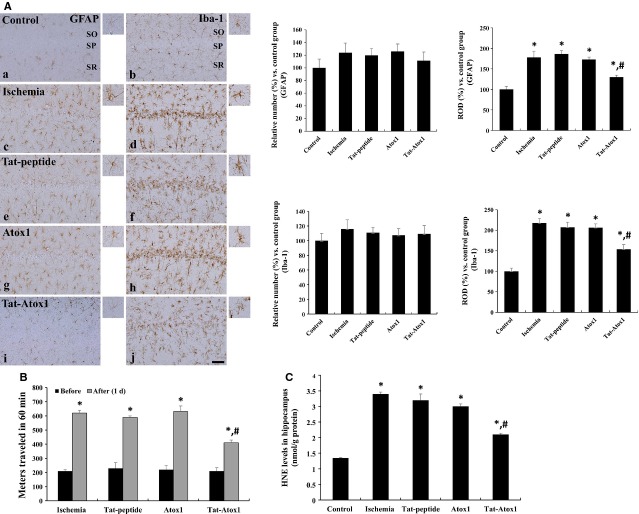
Inhibitory effects of transduced Tat-Atox1 protein in ischaemic animal models. Immunohistochemistry for GFAP (a, c, e, g and i) and Iba-1 (b, d, f, h and j) in the CA1 region of the control (a and b), ischaemia (c and d), Tat peptide (e and f), Atox1 protein (g and h) and Tat-Atox1 protein-treated (i and j) groups 4 days after ischaemia/reperfusion (A). SP, stratum pyramidale; SO, stratum oriens; SR, stratum radiatum; bar = 50 μm. The locomotor activity in gerbils before and 1 day after ischaemia-reperfusion in ischaemia, Tat peptide, Atox1 protein and Tat-Atox1 protein-treated groups. Spontaneous locomotor activity is evaluated in terms of entire distance (metres) travelled before and 1 day after ischaemia-reperfusion (B) (*n* = 5 per group; *P < 0.05, significantly different from the before group, ^*#*^*P* < 0.05, significantly different from the ischaemia group). The bars indicate standard error (SE). Analysis of HNE levels in the hippocampus of control, ischaemia, Tat peptide, Atox1 protein and Tat-Atox1 protein-treated groups at 3 hrs after ischaemia-reperfusion (C) (*n* = 5 per group, *P < 0.05, significantly different from the control group, ^*#*^*P* < 0.05, significantly different from the ischaemia group). The bars indicate SE.

Glial fibrillary acidic protein-immunoreactive astrocytes have been identified as thread-like bodies with small cytoplasm 44,45. GFAP-immunoreactivity steeply increases when astroglial cells are damaged by gliosis. Figure6A (left side) represents the effects of Tat-Atox1 protein on ischaemia-induced gliosis, which was indicated by GFAP-immunoreactive astrocytes in all strata (SP: stratum pyramidale; SO: stratum oriens; SR: stratum radiatum) of the CA1 region. In the ischaemia groups, GFAP-immunoreactive astrocytes exhibited abnormal thorn-shaped and thread-like morphology in the hippocampal CA1 region along with a large amount of cytoplasm. The morphology of GFAP-immunoreactive astrocytes did not differ significantly in either the Tat peptide or the Atox1 protein groups from that of the ischaemia groups. In the Tat-Atox1 protein treated ischaemia group, few GFAP-immunoreactive astrocytes featuring abnormal thorn-shaped bodies and cytoplasm were found while the remaining astrocytes had a thread-like morphology with thorn-shaped bodies and a small amount of cytoplasm.

To assess the effects of Tat-Atox1 protein against ischaemia-induced gliosis, the immunohistochemistry of microglial marker Iba-1 in the hippocampal CA1 region was assessed (Fig.6A, right side). Iba-1 immunoreactive microglia were observed in the SO and SR in the untreated control group. Iba-1-immunoreactive microglia exhibited morphological features of round-shaped cytoplasm and thread-like thorn-shaped bodies. Iba-1-immunoreactive microglia was observed in not only the SO and SR but also in the SP in the ischaemia group. In the SP, abnormally developed cytoplasms without thorn-shaped bodies were detected while abnormal cytoplasm featuring thick and shrunken thorn-like bodies were observed in the remaining regions. Iba-1-immunoreactive microglia cells in the massive layers of lipid indicate very active immune response. Iba-1-immunoreactive microglia morphology and distribution in the Tat peptide or Atox1 protein groups exhibited a similar pattern with those of the ischaemia group. A small amount of Iba-1-immunoreactive microglia were observed in the SP region in the Tat-Atox1 protein group, with cells composed of thick and shrunken thorn-shaped and thread-like, thorn-shaped cytoplasm. This indicates that the inflammatory response of the Iba-1-immunoreactive microglia cells was significantly diminished in the CAI region.

### Effects of Tat-Atox1 protein against locomotor activity and lipid peroxidation

To determine whether transduced Tat-Atox1 protein inhibits ischaemia-induced hyperactivity, we measured locomotor activity before and 1 day after ischaemia. Locomotor activity was 2.9 times higher in the ischaemia-reperfusion group, compared with the pre-ischaemia-reperfusion group (Fig.6B). The level of locomotor activity in the Tat peptide or Atox1 protein group was similar to that of the ischaemia group. The locomotor activity level was 1.9 times higher after pre-treatment with Tat-Atox1 protein.

To determine the inhibitory effects of transduced Tat-Atox1 protein on ischaemia-induced lipid peroxidation, we measured the level of HNE, a maker of lipid peroxidation, in the hippocampus. As shown in Figure6C, the HNE level was 1.35 nmol/g proteins in the control group. The HNE level increased 3 hrs after ischaemia-reperfusion in all experimental groups, compared with the control group. In the ischaemia group, the HNE level was 2.51 times higher that of the control group. The HNE levels of the Tat peptide and Atox1 protein groups were similar to that of the control group. A significant decline in the HNE protein level was observed in the Tat-Atox1 protein group, compared with the ischaemia group at 1.55 times that of the control group.

## Discussion

Atox1 protein has antioxidant functions in cells, protecting against damage by detoxifying cells under oxidative stress. Furthermore, Atox1 protein plays other important roles in cells including iron homoeostasis, cellular respiration, and antioxidant defence 1,6,8. In this study, we constructed a cell-penetrating Tat-Atox1 protein to investigate the protective effects of Atox1 against oxidative stress-induced neuronal cell death and ischaemic insult animal model.

Protein transduction domains are small peptides with the ability to deliver biologically active molecules into tissues, including cell lines and the brain 16–19. Many studies have suggested the therapeutic potential of protein transduction into cells and tissues 25,27,31,46–48. However, a number of factors affect the potential therapeutic benefits of PTD fusion protein transduction including the nature of the target protein, the cell type and the type of PTD used 17. As the transduction mechanism is not yet fully understood, the precise transduction mechanism of PTD fusion proteins requires further study.

In this study, we assessed the dose- and time-dependent transduction of purified Tat-Atox1 protein into cells using Western blotting. By using DAPI and Alexa fluorescence, we further confirmed that Tat-Atox1 protein effectively traversed across the HT-22 cell membrane and localized in the cells. Thus, our findings demonstrate the intracellular transduction of PTD fusion protein without cell-surface binding.

Reactive oxygen species damage cellular macromolecules including proteins, lipids and nucleic acids 49. We examined the roles of transduced Tat-Atox1 protein on cell viability in H_2_O_2_-stimulated cells. After exposure to 1 mM H_2_O_2_, 43% of cells survived. Cell survival increased to up to 78% in Tat-Atox1 protein pre-treated cells. We also assessed the antioxidant action of transduced Tat-Atox1 protein against H_2_O_2_-induced oxidative stress using ROS sensitive dye DCF-DA. As DNA fragmentation is induced by various factors such as oxidative stress, the effects of transduced fusion protein on DNA fragmentation were measured by TUNEL staining. The amount of TUNEL-positive cells was significantly higher in the H_2_O_2_-stimulated cells, compared with the control cells. The results of this study confirmed that the transduced Tat-Atox1 protein has an anti-apoptotic defensive effect against oxidative stress-induced cell death. The anti-apoptotic effects of transduced protein are consistent with the results which concluded that transduced Atox1 protein had an antioxidant defensive effect against superoxide and H_2_O_2_ toxicity. We therefore suggest that transduced Atox1 protein provides protection to cells against a variety of oxidative stress including damage to hydroxyl radicals, DNA damage and scavenges superoxide radicals.

Many studies are underway to investigate the effects of oxidative stress and excess ROS exposure on functional damage in the mitochondrial membrane, including membrane decomposition. Mitochondrial membrane damage has an important impact on mitochondrial function in mammalian cells. The apoptotic activator (Bax) and apoptotic inhibitor (Bcl-2) play essential roles in apoptosis 32,33. On the basis of the results of this study, we propose that Tat-Atox1 protein increased Bcl-2 expression and protected cells from H_2_O_2_-induced neurotoxicity. Tat-Atox1 protein also exhibited protective defence against ROS-induced apoptosis in H_2_O_2_-stimulated cells. The protective actions of transduced Tat-Atox1 protein against H_2_O_2_-induced cell death were identified by reduced expression of apoptotic proteins such as Akt and Bad. Recently, studies showed that PI3K/Akt pathway is involved in the adaptive effect of H_2_O_2_ pre-conditioning in various cell types 38–40. Thus, we examined the effect of H_2_O_2_ pre-conditioning against cell viability and activation of Akt. Cell viability was partially increased and phosphorylation of Akt was also increased in the H_2_O_2_ pre-conditioning. However, transduced Tat-Atox1 protein did not effect on the H_2_O_2_ pre-conditioning (data not shown). To understand the precise antioxidant effect of transduced Tat-Atox1 protein on H_2_O_2_ preconditioning induced cell of transduced Tat-Atox1 protein in the H_2_O_2_ pre-conditioning, further study is needed to elucidate. We also examined the effects of Tat-Atox1 protein on H_2_O_2_-induced ROS and discovered diminished levels of p53 protein expression as well as the provision of inhibitory activation of the proteins in response to H_2_O_2_-induced ROS.

It is well-documented that the oxidative-induced neuronal cell death mechanism is associated with the phosphorylation of MAPKs 41–43. We evaluated the relationship between transduced Tat-Atox1 protein and H_2_O_2_-induced oxidative stress. Thus, the activation of MAPKs, p38, JNK and ERK were measured after exposure to H_2_O_2,_ resulting in oxidative stress. Tat-Atox1 protein inhibited the activation of these MAPKs, demonstrating the antioxidant defensive effect of Tat-Atox1 protein.

Oxygen deprivation after brain ischaemia-reperfusion causes ischaemic injury 50. Although the mechanism by which ischaemia causes neuronal damage is not understood, it is evident by the ischaemic neuronal damage associated with ROS-induced neurodegeneration and subsequent increases in intracellular oxidative stress 51,52. To determine the effects of Tat-Atox1 protein on transient ischaemic neuronal damage, we intraperitoneally injected gerbils with Tat-Atox1 protein 30 min. after ischaemia onset. Tat-Atox1 protein was transduced into the CA1 region traversing the BBB. In addition, immunohistochemistry showed that Tat-Atox1 protein significantly protected against CA1 neuronal cell death in animal ischaemia models.

NeuN is a neuronal nuclear antigen that is commonly used as a biomarker for neurons. NeuN immunoreactivity has been widely used to identify neurons in tissue culture and sections of brain tissue 21,30. A greater number of NeuN-positive neurons were observed in the hippocampal CA1 regions of the Tat-Atox1 protein group, compared with the Tat peptide-treated group. Because only the Tat-Atox1 protein group demonstrated decreased neuronal damage following ischaemic injury in the hippocampal CA1, we concluded that Tat-Atox1 protein has a protective effect against neuronal cell death.

Damage to brain or spinal cord cells by injury or diseases, can cause astroglial cells to react by rapidly producing more glial fibrillary acidic protein 44,45. In our experiment examining the effects of Tat-Atox1 protein on GFAP-immunoreactive astrocytes in response to ischaemic gliosis, reduction in GFAP-immunoreactive astrocytes was most significant in the Tat-Atox1 protein group, compared with other experimental groups.

Specifically expressed in microglia, Iba-1 protein is up-regulated during the activation of these cells. Following nerve injury, central nervous system ischaemia, and brain diseases this expression is up-regulated 53. We also identified the effects of Tat-Atox1 protein on ischaemia-induced gliosis using the microglial marker Iba-1-immunoreactive microglia. So, Iba-1-immunoreactive microglia is responsible for immune responses and removal of radicals. The cells become activated under certain conditions while they usually remain in a resting state. Iba-1 expression was less significant in response to ischaemia in the Tat-Atox1 protein group, compared with the Tat peptide or the Atox1 protein group. Iba-1 expression was prominent in the SP region in the Tat peptide or the Atox1 protein groups. These results indicate that Tat-Atox1 protein significantly protects against ischaemic injury by decreasing the activation of astrocytes and microglia.

On the basis of the results of earlier studies reporting increases of animal locomotor activity in reaction to ischaemia-reperfusion 30,54, we assessed the effects of Tat-Atox1 protein on locomotor activity. In this study, a reduction in locomotor activity was observed in the Tat-Atox1 protein group. Also, we discovered an increase in HNE levels after ischaemia onset. However, HNE levels decreased in the Tat-Atox1 protein group. Our results clearly show that Tat-Atox1 protein protects against ischaemic injuries resulting from oxidative stress and acts as an antioxidant. Therefore, we propose the potential efficacy of Tat-Atox1 protein as a therapeutic agent for the treatment of a variety of neurodegenerative diseases.

In conclusion, we achieved the efficient transduction of Tat peptide fused human Atox1 protein *in vitro* and *in vivo* and proved the protective effects of Tat-Atox1 on oxidative stress-induced cell death and ischaemic insults. Although further studies are needed to explore more specific mechanisms, this novel fusion protein represents a potential new therapeutic strategy against ischaemic damage as well as for the treatment of ROS-induced diseases, including stroke.
